# Usefulness and limitations of convergent cross sorting and continuity scaling methods for their application in simulated and real-world time series

**DOI:** 10.1098/rsos.221590

**Published:** 2023-07-12

**Authors:** Adolfo D. Bahamonde, Rodrigo M. Montes, Pablo Cornejo

**Affiliations:** ^1^ Interdisciplinary Center for Aquaculture Research (INCAR), University of Concepción, O’Higgins 1695, Concepción, Chile; ^2^ Mechanical Engineering Department, University of Concepción, Concepción, Chile

**Keywords:** causality, convergent cross sorting, continuity scaling, mutual information

## Abstract

Causality detection methods are valuable tools for detecting causal links in complex systems. The efficiency of continuity scaling (CS) and the convergent cross sorting (CSS) methods to detect causality was analysed. Usefulness and limitations of both methods in their application to simulated and real-world time series was explored under different scenarios. We find that CS is more robust and efficient than the CSS method for all simulated systems, even when increasing noise levels were considered. Both methods were not able to infer causality when time series with a marked difference in their main frequencies were analysed. Minimum time-series length required for the detection of a causal link depends on intrinsic system dynamics and on the method selected to detect it. Using simulated time series, only the CS method was capable to detect bidirectional causality. Causality detection, using the CS method, should at least include: (i) causality strength convergence analysis, (ii) statistical tests of significance, (iii) time-series standardization, and (iv) causality strength ratios as a strength indicator of relative causality between systems. Causality cannot be detected by either method in simulated time series that exhibit generalized synchronization.

## 1. Introduction

A fundamental question when studying real-world complex systems is how to establish cause–effect relationships between two time-dependent variables (e.g. *x* → *y*). The identification of causal links between variables that affect the system’s behaviour to some degree is vital for the development of reduced-order models (ROMs) and control systems [1], as well as for the identification of governing equations [2]. Causality detection is particularly important in health research, where causality detection methods have been used to identify the direction of brain–heart interactions [3,4] and to detect interactions between different parts of the brain [5]. Recently, causality analysis was employed to study the spread of the COVID-19 pandemic [6,7] and its effects on stock markets [8]. Another research area in which causality identification and quantification are important is in the Earth and Atmospheric Sciences. Here, the identification of causal interactions allows for the improvement of the predictive capacity of climate models [9], the determination of causal relationships in the study of climate change [10] and its consequent social effects [11].

In its most fundamental form, a causal relationship appears as a term in the differential equations describing the system’s temporal evolution. However, when studying real-world systems, it is common to have no access to these equations, but rather to time series obtained through measurements. Different methods have been developed to identify causal connections between variables (e.g. *x* and *y*), which can be classified into two large classes: statistical methods based on information theory [12], and those based on the reconstruction of the system’s phase space [13]. Granger causality [14], one of the first proposed quantities to study causality, belongs to the first class. This latter method was used to estimate direct causal relationships in ecological systems [15,16].

Subsequently, other methods and quantities were developed, such as transfer entropy-based methods [17], compression complexity [18], embedding entropy [19], PCMCI [20] and methods based on generalizations of conditional mutual information [1]. The second class, and the one we will focus on in the present study, was developed over the last decade and is based on Taken’s theorem [21]. The second large class, which is the focus of the present study, is deeply rooted on Takens’ theorem, and assumes that the attractor of the system can be recovered from analysed time series [21–25]. Probably, the most well-known method of this class is convergent cross mapping (CCM) proposed by Sugihara *et al*. [26]. Subsequently, different modifications of the CCM method were proposed for the identification of causal relationships in systems with time delays [27] or by using other causality indicators such as pairwise asymmetric inference [28]. However, up to date, one of the best-performing modifications introduced to the CCM method is known as convergent cross sorting method (CCS) [29], which uses a dimensionless ranking to order points in phase space, making the method independent of geometric transformations that distort the distance and preserve the relative order (multiplication or addition of some constant to the time series).

Recently, an approach to identify causal relationships based on the continuity condition for mapping between the reconstructed attractors was proposed. This condition has previously been used to determine the reconstruction parameters of attractors [30,31]. To the best of our knowledge, the continuity condition was first proposed as a measure of causality in [13]. The continuity scaling (CS) method [32] was recently formalized, and a measure of causal strength was established in terms of the continuity condition, allowing the systematization of its application.

The proposal of several methods to detect causality over a short time period shows not only the relevance of this research area but also its fast development rate over last years. However, since these methods are still in the early stages of maturity, their performance can be significantly impacted under different conditions, leading to considerable variability in their results. In particular, in Earth Sciences, where time series can be characterized by seasonality (dominant low frequencies), bidirectional interactions of different intensities (synchronization can occur), there may be indirect causalities (in biology, and it is common to find feedback loops or causal chains [33,34]), we usually find short time series of few measurements (*n* ∼ 200−500), series containing different time scales (e.g. phenomena of interdecadal variability, such as El Niño–Southern Oscillation (ENSO) interacting with processes of synoptic variability) and noisy time series.

In this scenario, the available methods proposed to detect causality should be tested not only with time series generated using physical models (e.g. coupled logistic maps (LM), Rössler systems), but also with experimental and real-world time series to characterize them under multiple conditions where the performance of these methods can be seriously affected. In this study, we analyse different cases considering systems with complete synchronization and generalized synchronization, in the latter case the time series are not necessarily similar but are connected by a functional relationship [35]. Accordingly, the main objectives of this study are: (i) to determine the conditions under which CS [32] and CCS [29] methods can be safely applied to detect causality in real-world time series, (ii) to highlight the advantages and limitations of both methods for scientists within the Ecology, Biology, Earth and Marine Science communities, among others, who can use these methods to detect causal networks.

## 2. Causality detection methods

### 2.1. Convergent cross sorting method

CCS was recently proposed in [29], and its performance appears to be better than the method from which it was developed: CCM. A concise description of CCM is provided in the following paragraphs, and further details can be obtained from [26,27,36]. The CCM method proposes that if a variable *x*_*t*_ causally affects another variable *y*_*t*_, it is therefore possible to approximate *x* from *y*. This means that the information contained in *y* alone can be used to reconstruct past values of *x*, or in other words, time-delay embedding allows to detect the amount of information about *x* that has been encoded into *y* [26]. If we have access to some variables measured at time *t*, such as *x*_*t*_ and *y*_*t*_, those can be used to reconstruct two versions of the attractor $L_{x}$ and $L_{y}$, then we can reconstruct *x* from *y* and a cross-map exists from $L_{y}$ to $L_{x}$. In this context, the ‘causal effect that *x* has on *y*’ is quantified by how well *y* cross-maps *x* (they belong to a common dynamical system) [22,26,37]. The causal strength is quantified by the correlation between the original variable and the reconstructed variable, $\rho (x_{t},{\hat{\;f}}_{x_{t}}^{-1}y_{t}))$. However, Sugihara *et al*. [26] suggest another condition: in addition to the value of the correlation *ρ* there must be a convergence of its value time-series length increases. For the reconstruction of time series, the CCM method estimates ${\hat{x}}_{t}={\hat{\;f}}_{x_{t}}^{-1}(y_{t})$ as follows: considering a time *t*, associated with a point *X*_*t*_ in the attractor, the *d*_*x*_ + 1 nearest neighbours are searched (with *d*_*x*_ the embedding dimension). Then, the nearest neighbours are sorted according to their Euclidean distance to *X*_*t*_ and obtain times $t_{1}\cdots t_{d_{x}+1}$ associated with points $X_{1},\ldots ,X_{d_{x}+1}$ on the attractor. Then the variable ${\hat{y}}_{t}$ is recreated as ${\hat{y}}_{t}=\sum_{i=1}^{d_{x}+1}w_{i}y_{t_{i}}$. Where $w_{i}=e^{-(d(X(t),X(t_{i}))/d(X_{t},X_{t_{1}}))}$ and *d*( · , · ) denotes the Euclidean distance. This latter operation causes several problems with the CCM method mentioned in [29,37–39], the most relevant being:

(i) Requires relatively long time series to obtain reliable results, i.e. length $n∼O(10^{3})$.
(ii) Failure in oscillatory variables that show a highly dominant or shared single frequency.
(iii) Poor performance for noisy and also for strongly coupled time series.
(iv) Shows some problems when applied to time series that are not ‘fully deterministic’.

Short, oscillatory and noisy time series that are somewhere in between the extremes of being ‘fully deterministic’ (i.e. no measurement or process noise) and ‘fully stochastic’ (i.e. independently identically distributed noise) are ubiquitous in nature, making difficult the widespread use of CCM in geophysical, biological and ecological research [37,39]. The CCS method addresses some of these drawbacks by replacing the use of nearest neighbours based on Euclidean distance with a dimensionless distance ranking. The fundamental improvement is that, after estimating the distance between the reconstruction points, $d(X_{t_{i}},X_{t_{\;j}})$, those are sorted from the smallest to the largest and then replaced by a dimensionless ranking *R* consisting of evenly spaced values between 0 and 1. Finally, these ranking values, are sorted in the same order as the distances $d(X_{t_{i}},X_{t_{\;j}})$. To estimate causality, the ranking *R* is sorted again in the same way as the distance of the points in the space $L_{y}$, this is denoted by *R*_*x*_(*I*_*i*_). Then the error between the original rankings *R* and the reordered ranking *R*_*x*_(*I*_*i*_) is estimated. In this method, causality is basically a measure of the difference between both rankings. This error is normalized by the error that would be obtained if the rankings were randomly sorted. We call this function *E*(*R*) and it is approximated according to *E*(*R*) = *a* + *be*^*cR*^. The causality score given by the CCS method is the value *E*(0) [29].

The advantage of this procedure is that causality is independent of certain geometric transformations of the system variables. For example, the same result is obtained regardless of whether a standardized variable is used, which is a problem found when other methods are used.

### 2.2. Continuity scaling method

This method was proposed in [32] and is described in the following paragraph. Let us consider a dynamic system of two variables *x*_*t*+1_ = *f*(*x*_*t*_, *y*_*t*_), *y*_*t*+1_ = *g*(*y*_*t*_, *x*_*t*_). If we have access to time series of these variables, we can reconstruct the system’s attractor for lags (*τ*_*u*_, *τ*_*v*_) and embedding dimensions (*d*_*u*_, *d*_*v*_). The evolution of these two variables can be described using the functions $\hat{\;f}(x_{t},y_{t})$ and $\hat{g}(y_{t},x_{t})$. If we consider ${\hat{\;f}}_{x_{t}}(\cdot )=\hat{\;f}(x_{t},\cdot )$, we see that the role of this function is to map the variable *y*_*t*_, which lives in its reconstructed space $L_{y}$ to the space of *x*_*t*_, $L_{x}$. Similarly, ${\hat{\;f}}_{x_{t}}^{-1}(\cdot )$ performs the inverse operation. What was proposed in [32] means that if causality exists, there is a continuous map between the two spaces ($L_{y}$ and $L_{x}$). Thus, if we consider a neighbourhood in $L_{x}$ of radius $\epsilon_{x}$ that maps to the space $L_{y}$ using inverse function ${\hat{\;f}}_{x_{t}}^{-1}(\cdot )$, we should obtain a neighbourhood in $L_{y}$ whose radius *δ*_*y*_ must be an increasing function of $\epsilon_{x}$. In other words, continuity implies that as the size of the neighbourhood $\epsilon_{x}$ increases, the size of the pre-image of this neighbourhood (*δ_y_*) must also increase. Moreover, it should scale as $\delta_{y}∼\log(\epsilon_{x})$. In [32], the authors proposed the scaling of *δ*_*y*_ as a function of $\epsilon_{x}$ (value of the slope in logarithmic scale) as a measure of causal strength.

## 3. Causality detection in simulated and real-world time series

### 3.1. Logistic map

Coupled LMs are one of the most frequently used systems in causality studies [26,29,40]. This corresponds to a discrete system, whose dynamics are given by equation (3.1), which accounts for the interaction between two chaotic systems. The system exhibits synchronization for high values of coupling strength.

3.1 $$x_{1}(t+1)=x_{1}(t)[r_{1}-r_{1}x_{1}(t)-C_{1}x_{2}(t)]$$

and

3.2 $$x_{2}(t+1)=x_{2}(t)[r_{2}-r_{2}x_{2}(t)-C_{2}x_{1}(t)]\;.\;$$

The coupling is given by *C*_*i*_. In this study, we set *r*_1_ = 3.6 and *r*_2_ = 3.7 in the case of two coupled systems.

**Figure 1.**
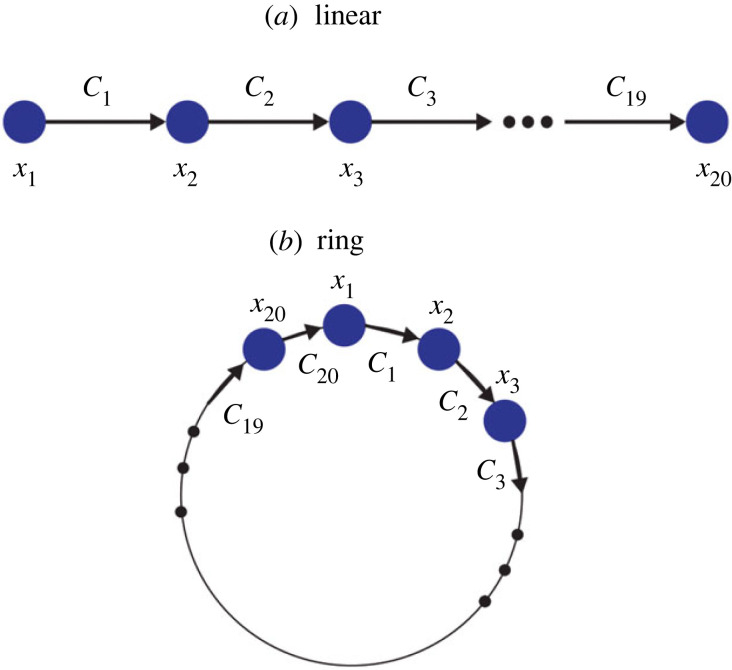
Arrangements considered. (*a*) Linear array with unidirectional causality. (*b*) Circular arrangement.

The chains of LMs coupled in networks with linear and ring topologies were also studied, as shown schematically in figure 1*a*,*b*. This allows us to investigate the method’s ability to detect direct causality and its potential failure in the presence of indirect causal relationships. In these latter cases, 20 different LMs were coupled, with the coupling constant taking uniformly spaced values in *C* ∈ {3.6, 3.8}.

### 3.2. Rössler–Lorenz system

The second system considered for causality analysis is the Rössler–Lorenz (RL) coupled system, which is presented and described in [41,42]. The governing equations for this system are the following:

3.3 $${\dot{x}}_{1}=-a(x_{2}+x_{3}),$$

3.4 $${\dot{x}}_{2}=a(x_{1}+0.2x_{2}),$$

3.5 $${\dot{x}}_{3}=a(0.2+x_{3}(x_{1}-5.7)),$$

3.6 $${\dot{y}}_{1}=10(-y_{1}+y_{2}),$$

3.7 $${\dot{y}}_{2}=28y_{1}-y_{2}-y_{1}y_{3}+Cx_{2}^{2}$$

3.8 $$\;\mathrm{and}\;{\dot{y}}_{3}=y_{1}y_{2}-\frac{8}{3}y_{3}.$$

**Figure 2.**
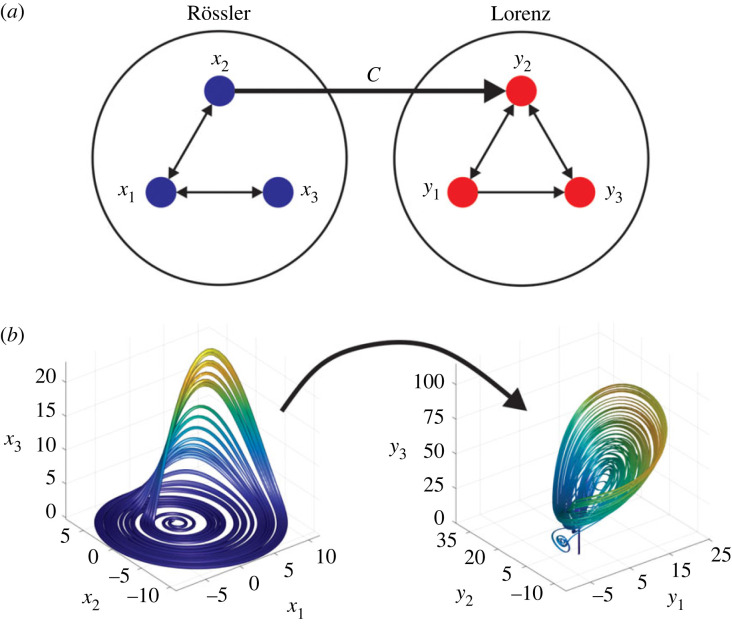
Coupled Rössler–Lorenz system. (*a*) Causal relationships between the variables. (*b*) Attractors generated by the system.

The equations show that the coupling is of the form *x*_2_ → *y*_2_ through the constant *C*, as shown schematically in the causal network in figure 2*a*. This system allows us to study the effectiveness of CCS and CS methods in a continuous, chaotic system (the attractors are shown in figure 2*b*). This system also exhibits generalized synchronization, indicating that an invertible functional relationship exists between the subsystems [43]. This occurs for C≳2 when *a* = 6 and in the ranges 2.1 ≤ *C* ≤ 2.7 or *C* > 2.9 when *a* = 10 [42].

### 3.3. Rössler–Rössler system

The third system under study consists of two coupled Rössler systems. The equations that describe the evolution of the Rössler–Rössler (RR) system are

3.9 $${\dot{x}}_{1}=-\omega_{1}x_{2}-x_{3},$$

3.10 $${\dot{x}}_{2}=\omega_{1}x_{1}+a_{1}x_{2},$$

3.11 $${\dot{x}}_{3}=x_{3}(x_{1}-c)+b_{1},$$

3.12 $${\dot{y}}_{1}=-\omega_{2}y_{2}-y_{3}+C(x_{1}-y_{1}),$$

3.13 $${\dot{y}}_{2}=\omega_{2}y_{1}+a_{2}y_{2}$$

3.14 $$\;\mathrm{and}\;{\dot{y}}_{3}=y_{3}(y_{1}+c)+b_{2}.$$

**Figure 3.**
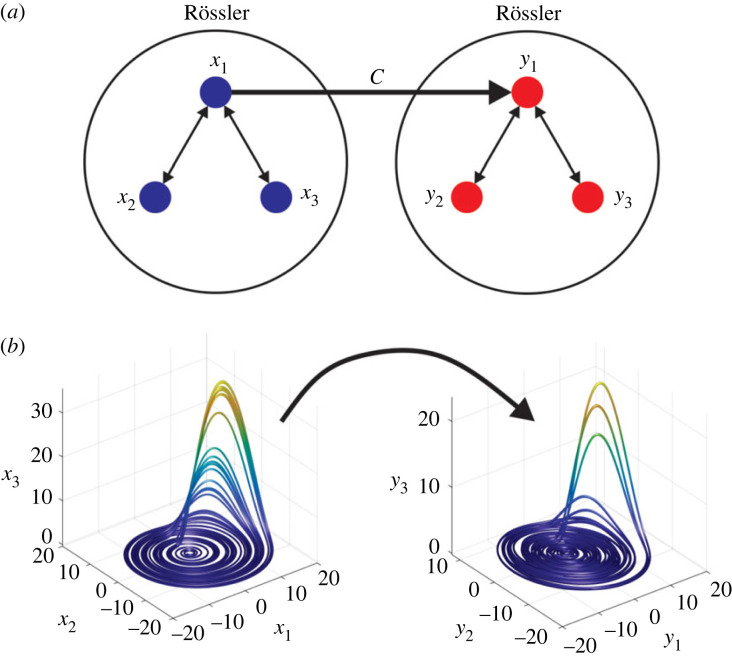
Coupled Rössler–Rössler system. (*a*) Causal relationships between the variables. (*b*) Attractors generated by the system.

This system has been previously studied in the context of causality detection [18]. We see that the direct coupling corresponds to *x*_1_ → *y*_1_, as shown in the causal network in figure 3*a*. The advantage of considering this system is that parameters *ω*_*i*_ (*i* = 1, 2) in the RR equations are associated with the main frequencies of the variables. This property allowed us to study the effect of different frequency ratios (*r* = *ω*_1_/*ω*_2_) on the performance of both causality detection methods.

### 3.4. Real-world time series

**Figure 4.**
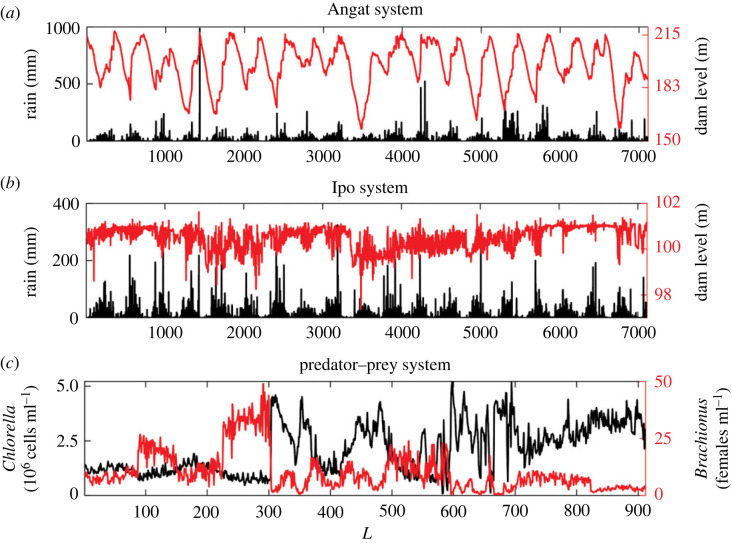
Time series of rainfall (mm) and dam water level (m) in (*a*) Angat and (*b*) Ipo dams [44]. (*c*) *Brachionus* (females ml^−1^) and *Chlorella* (cells ml^−1^) concentration time series [45].

We applied the previously described causality detection methods to six time series from three real-world systems shown in figure 4. These correspond to the rainfall-dam level systems for the Angat (figure 4*a*) and Ipo (figure 4*b*) dams, both located in the Philippines [44]. The second system (figure 4*c*) corresponds to a predator–prey system cultured in a chemostat described in [45] and available in [46], characterized by the planktonic rotifer *Brachionus calyciflorus* that feeds on the unicellular green algae *Chlorella vulgaris*, resulting in oscillations of species populations in different experimental trials. This system was controlled by adjusting the dilution rate and the addition of Nitrogen (N), which can limit the algal growth. The N concentration determines the birth rate of *Chlorella*, and the concentration of this species determines the birth rate of *Brachionus* [45].

**Figure 5.**
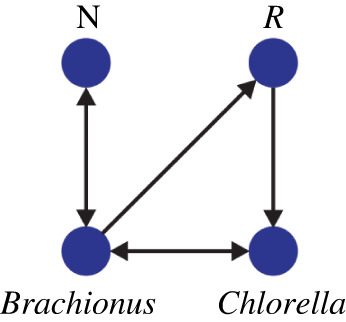
Causal network associated with the predator–prey mathematical model proposed in [45]. N and *R* correspond to the concentration of nitrogen and *Brachionus* in the reproductive stage. Here, we only consider data for *Brachionus* and *Chlorella*.

In [45], a mathematical model for the *predator–prey* system is proposed, and it is illustrated in figure 5.

Time series under study have some desired characteristics to be tested using both causality detection methods. They are intermittent, and their main oscillation frequencies are associated with markedly different frequency bands. A particularly useful piece of information to test the ability of both causality detection methods is that the directions of causal links are known for both systems. In the rainfall–dam systems (Angat and Ipo), it goes from the rainfall to the water level. In the chemostat system, bidirectional causality for the concentrations of *Brachionus* and *Chlorella* is established as observed by their fluctuations over time (figure 4*c*) and its causal network (figure 5).

## 4. Results

### 4.1. Causality detection in simulated systems

**Figure 6.**
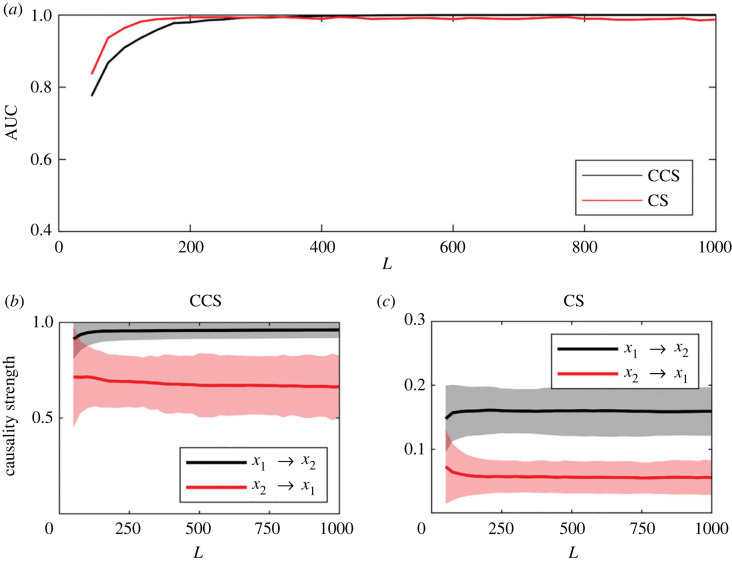
Effect of time-series length (*L*) on the efficiency of the methods in detecting and measuring causality strength for the logistic map system (*C*_1_ = 0, *C*_2_ = 0.1). The values were averaged over 1000 realizations with random initial conditions. (*a*) AUC. (*b*) CCS method. (*c*) Continuity scaling (CS) method.

Receiver operating characteristic (ROC) curves [47] were calculated, and the areas under the ROC curves (AUC) were used to estimate the efficiency of both causality detection methods in terms of different parameters. Results for the LM system are shown in figure 6. In figure 6*a*, it shows the variation of the AUC as a function of the time-series length. The AUC results for the CCS method coincide with the results reported in [29]. The CS method is more efficient for short time series, reaching AUC ≈ 1 in *L* ≈ 150, whereas for the CCS method the maximum efficiency is reached at *L* ≈ 200.

The lower panels of figure 6 show the causality strength detected by both methods for different time-series length in the correct (*x*_1_ → *x*_2_) and wrong (*x*_2_ → *x*_1_) directions.

**Figure 7.**
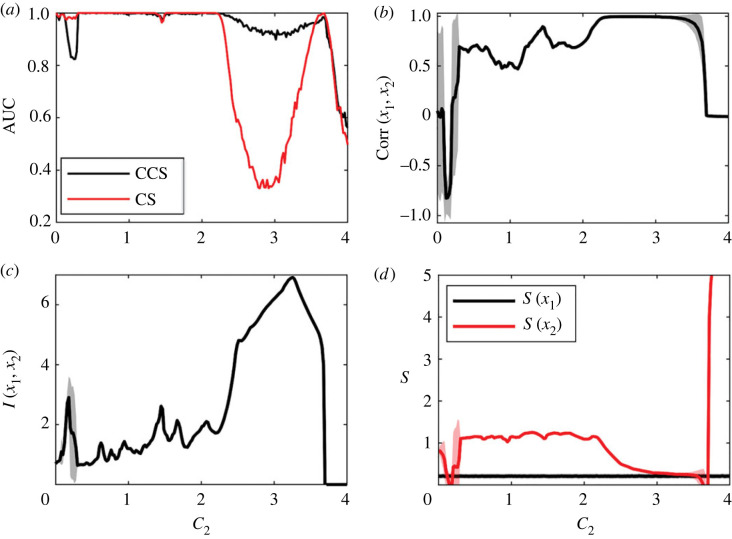
Results for the logistic map system. The values are averaged over 1000 realizations with random initial conditions. Time-series length was set to *L* = 400 and *C*_1_ was set to 0. (*a*) AUC as a function of coupling force. (*b*) Correlation between *x*_1_ and *x*_2_. (*c*) Mutual information between *x*_1_ and *x*_2_, estimated continuously with the Kraskov–Stögbauer–Grassberger (KSG) estimator [48]. (*d*) Entropy of the time series, estimated continuously with the Kozachenko–Leonenko (KL) estimator [49].

The effect of coupling strength on the efficiency of both methods is shown in figure 7*a*. For low values of *C*_2_, both methods have maximum efficiency. It starts to decrease at *C*_2_ ≈ 2.4 for the CS method and *C*_2_ ≈ 3.2 for the CCS method. At these coupling values, the systems begin to synchronize, as can be observed in the increase in mutual information (MI) and correlation (figure 7*b*,*c*).

**Figure 8.**
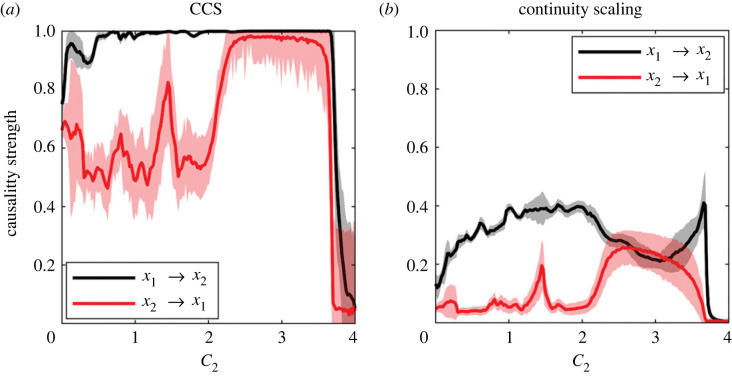
Detection of causality strength using both methods for the logistic map system (same data as in figure 7).

The coupling strength detected by each method is shown in figure 8. The causality detected by the CS method increases progressively as *C*_2_ increases, but this was not observed in the causality strength detected by the CCS method.

**Figure 9.**
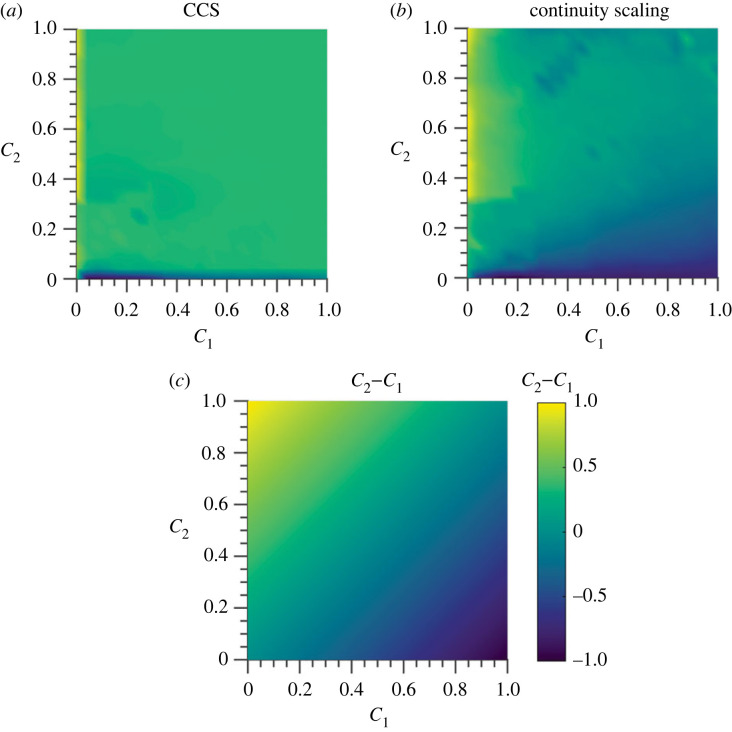
Results for bidirectionally coupled logistic maps. The *C*_1_ − *C*_2_ plane was discretized in a grid of 25 × 25 points between 0 and 1. The results for each point were averaged over 50 realizations. Time-series length is *L* = 400. (*a*) Difference in detected causal strength using CCS method. (*b*) Difference in detected causal strength using CS method. (*c*) Coupling difference for logistic maps.

The ability of the methods to identify bidirectional interactions is evaluated in the results of figure 9, which shows the results for the general bidirectional case. In the three figures, we display the difference between the causality strengths detected in the two directions for the CCS (figure 9*a*) and CS (figure 9*b*) methods, while the actual difference between the couplings is shown in figure 9*c*.

In table 1, two statistical measures of efficiency are shown. The error corresponds to the root-mean-square error (RMSE), and the AUC is obtained calculating the *p*-value. We computed the *p*-value for each point in the grid with 100 surrogates (for each realization) using the stationary bootstrap [50,51]. The points where the *p*-values were less than 0.05 are considered as statistically significant, meaning that the method detects causality at that point, in the direction considered.

**Table 1.** Statistical measures of efficiency for bidirectional causality detection in coupled logistic maps.

	**RMSE**			
	*C*_1_	*C*_2_	*C*_2_ − *C*_1_	**AUC**
CCS	0.544	0.563	0.450	0.5533
CS	0.307	0.272	0.297	0.962

**Figure 10.**
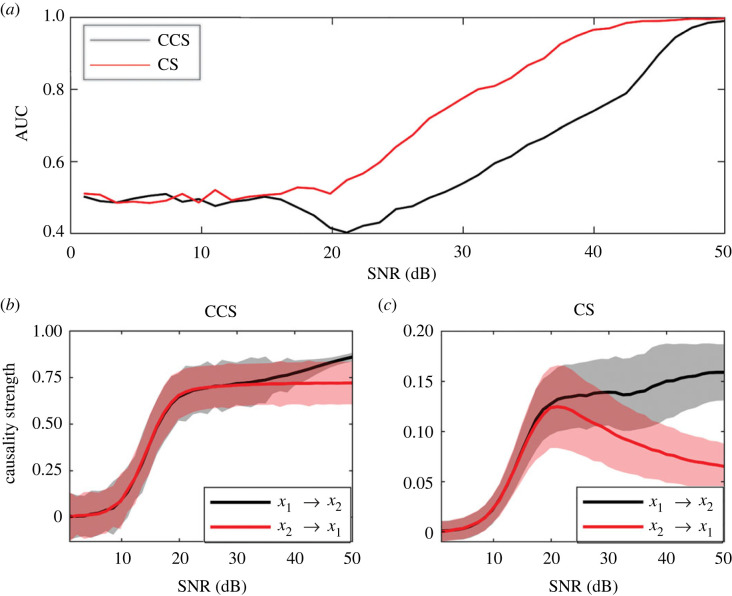
Effect of noise (dB) on the AUC for the logistic map. For all cases, time-series length is *L* = 400. The couplings were set to *C*_2_ = 0.1, *C*_1_ = 0. Signal-to-noise ratio (SNR) between 1 and 50. Results were obtained by averaging over 1000 realizations for each noise level. (*a*) Variation of AUC as SNR increases. Causality strength detected using the (*b*) CCS method and (*c*) the CS method.

The variation of the AUC with the increase in the time-series noise level, measured by the signal-to-noise ratio (SNR) is shown in figure 10*a*. When SNR ∼ 1, the noise amplitude is of the same order as the values of time series. As the SNR increases, the effect of noise on the time series decreases. Gaussian white noise was used in all the simulations. The effect of noise on the causality strength detected by CCS and CS methods are visible in figure 10*b*,*c*. The efficiencies of both methods for high noise levels (SNR≲15) are similar (approx. 0.5); however, the CS method surpasses the CCS method at SNR ≈ 18 (figure 10*a*). which can be clearly observed when causality curves separate from each other at this SNR level (figure 10*c*).

**Figure 11.**
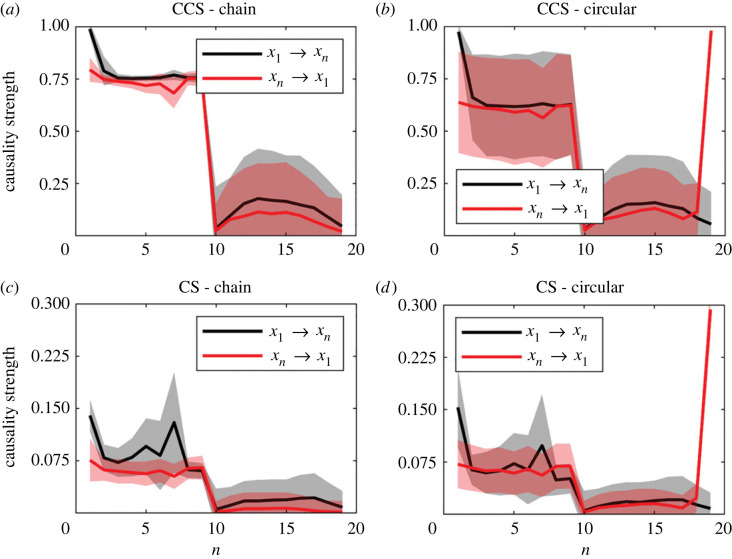
Detection of causality strength using CCS and CS methods for chain and circular causal arrays shown in figure 1. Results are averaged over 1000 simulations for each array. The time-series length is *L* = 400. (*a*) CCS method. (*b*) Continuity scaling method.

Causality strength detected by both methods for LM networks (figure 1) are shown in figure 11 for the CCS (figure 11*a*) and CS (figure 11*b*) methods.

**Figure 12.**
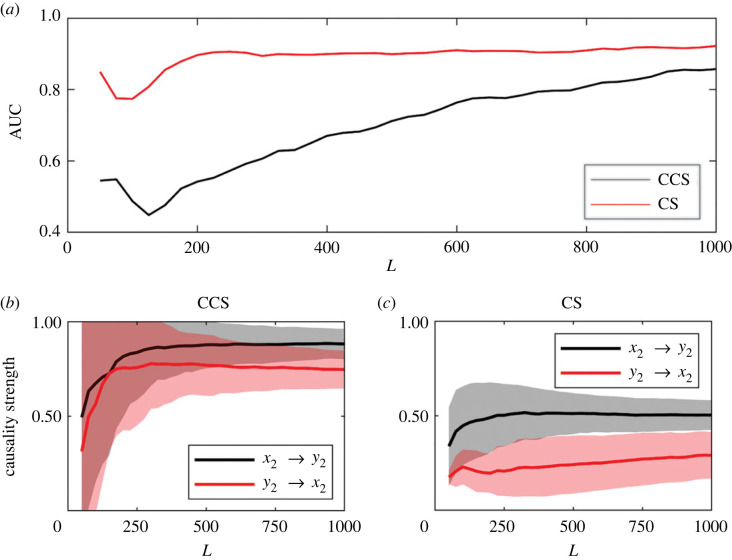
Effect of time-series length (*L*) on the efficiency of the methods for the Rössler–Lorenz (RL) system. Results were averaged over 1000 simulations with random initial conditions (*C* = 2). (*a*) Variation of the AUC as time-series length *L* increases. (*b*) Causality detected by the CCS method. (*c*) Causality detected by the continuity scaling (CS) method.

In the case of causality detection for the continuous RL system, there is only one direct causal relationship, *x*_2_ → *y*_2_, which is defined by the coupling constant *C*, as can be observed in figure 2*a*. Once again, the CS method outperforms the CCS method. For the former, AUC values over approximately 0.85 were obtained at reduced time-series lengths (*L* ≈ 200), as shown in figure 12*a*. By contrast, for the CCS method, AUC values over 0.85 were obtained at *L* ≈ 1000.

**Figure 13.**
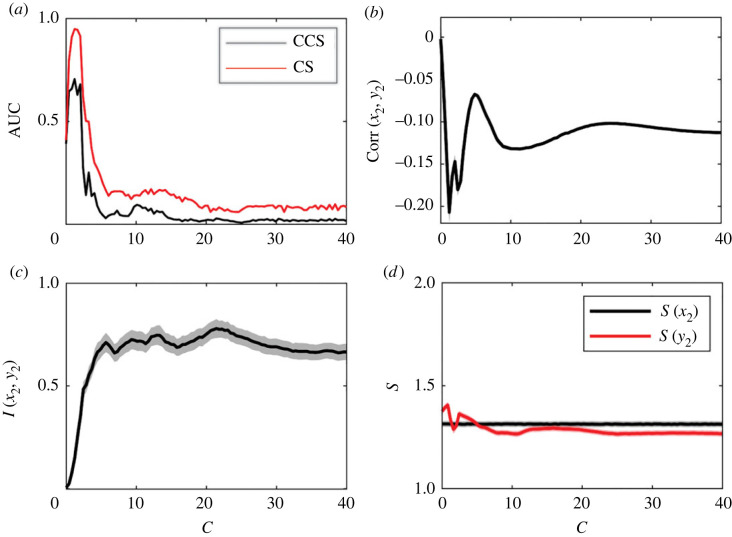
Effect of the coupling force on the efficiency of the methods for the RL system. Results were averaged over 1000 simulations with random initial conditions for each linkage value. The time-series length was set to *L* = 400. (*a*) Variation of AUC as coupling force increases. (*b*) Correlation between *x*_2_ and *y*_2_. (*c*) Mutual information between *x*_2_ and *y*_2_, estimated continuously with the KSG estimator. (*d*) Entropy of the time series estimated continuously with the KL estimator.

The effect of coupling strength (*C*) for both causality detection methods applied to the RL system can be observed in figure 13*a*. We note that the maximum efficiency for both methods is reached around *C* ≈ 2 (being the AUC greater for the CS than for the CCS method) and above this value a fast decrease in the AUC for both methods can be observed. The efficiency is approximately equal for high coupling values. The AUC is close to 0 for the CCS method between 20 < *C* < 40 and AUC ≈ 0.1 for the CS method for the same coupling strength range values. This latter result means that both methods are detecting a reverse causal relationship (*y*_2_ → *x*_2_) which clearly does not exist (figure 2*a*).

**Figure 14.**
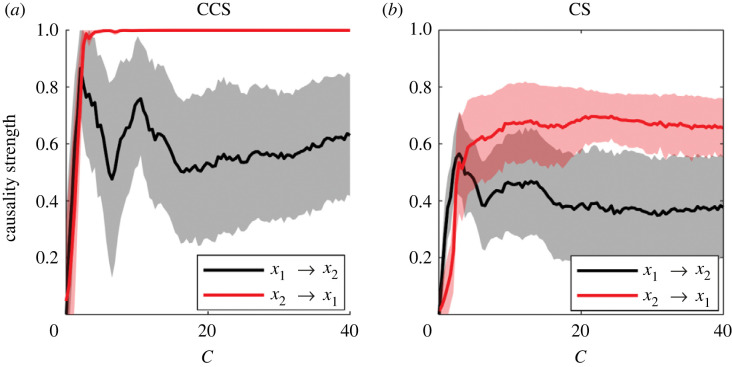
Detection of causality strength using both methods for the RL system (same data as in figure 13)

Figure 14 shows the causality strength detected by CS and CCS methods for different values of the coupling force *C* in the RL system.

**Figure 15.**
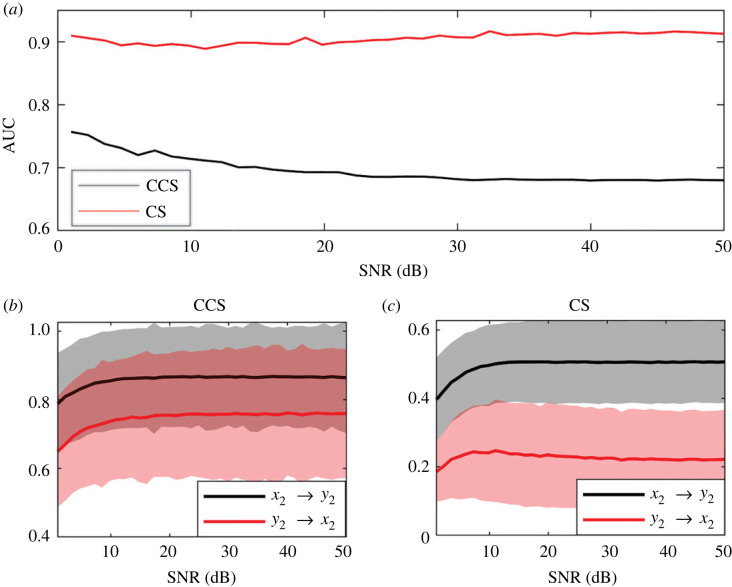
Effect of noise level (dB) on the efficiency of the methods for RL system. For all cases, time-series length was set to *L* = 400. The coupling force was fixed at *C* = 0.1. SNR varied between 1 and 50. The results were averaged over 1000 realizations for each noise level. (*a*) Variation of AUC as SNR increases. Causality strength estimated using the (*b*) CCS and (*c*) CS methods.

Figure 15*a* shows the variation in the AUC with noise level for both methods. AUC changes are smaller and not as marked as observed for the LM case (figure 10). In the case of the CCS method, the efficiency is higher when noise predominates. This is not observed in the CS method, which shows a slight improvement in its efficiency with noise reduction. We want to remark that the CS method yielded better results than the CCS method for all noise levels.

**Figure 16.**
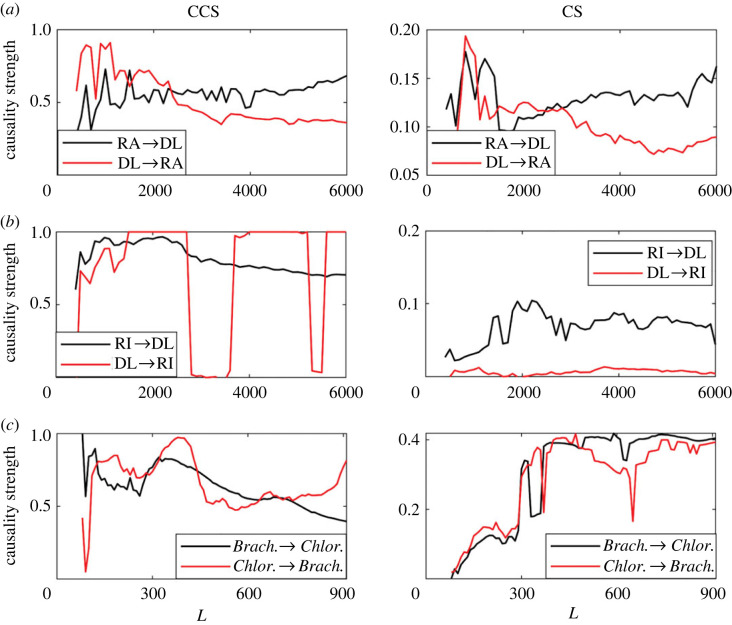
Efficiency of both methods for the LR system. Results were averaged over 1000 simulations with random initial conditions for each value of *r*. (*a*) Variation AUC as *r* increases. Causality strength detected by the (*b*) CCS and (*c*) CS methods.

The variability of the AUC as a function of the frequency ratio *r* = *ω*_1_/*ω*_2_ (see equation (3.9)) was analysed to detect the possible effect that differences in the main frequencies of the analysed signals can have on the ability of both methods to detect causality in the RR system (figure 16). The frequency *ω*_1_ was set to 1, and only *ω*_2_ was allowed to vary. A low *r* indicates a high *ω*_2_ frequency in comparison with *ω*_1_ (figure 16*a*) shows the efficiency of the methods in terms of the frequency ratio. For low *r* values, the efficiency is approximately 0. Thus, they detect a causal relationship from *y*_1_ → *x*_1_, which does not exists (figure 3*a*). The CS method was more efficient for all *r* values except in the cases where both methods fail: when both frequencies are the same (*r* = 1) and when the difference is large (*r* close to 0).

Table 2 shows a summary of our results for the three simulated systems using CCS and CS methods under different time-series length/noise level scenarios. It can be observed that the CS method outperforms the CCS method in all scenarios.

**Table 2.** Summary of results for simulated systems. A critical AUC value equal to 0.9 was considered. Cross marks indicate when AUC value was less than 0.9 or when non-monotonic variation without convergence was obtained.

	**CCS**		**continuity**	
	length^a^	noise	length^a^	noise
LM	>150	>46.23	>100	>37.4
RL	✗	✗	>225	>22.4
**frequency**				
	**CCS**		**continuity**	
RR	✗		*r* ≥ 1.5	

^a^A critical AUC value equal to 0.95 was considered for LM systems.

### 4.2. Causality detection in real-world time series

Table 3 shows the statistical significance of the detected causality in real-world time series for each method. Significance levels were calculated using the stationary bootstrap, which is recommended for time series with non-independent observations [50]. The mean block size was selected based on [51]. In total, 100 surrogates were created for each time series, after which a *p*-value test was performed. If causality was detected, its value was considered significant only if *p* ≤ 0.05.

**Table 3.** Statistical significance for causality detection in real-world systems using CCS and continuity scaling methods. RA, rain Angat system; RI, rain Ipo system; DL, water level of the respective system.

**system**		**CS**		**CCS**	
1	2	1 → 2	2 → 1	1 → 2	2 → 1
RA	DL-A	✓	✓	✓	✓
RI	DL-I	✓	✗	✓	✗
*Brach*.	*Chlor*.	✓	✓	✗	✓

**Figure 17.**
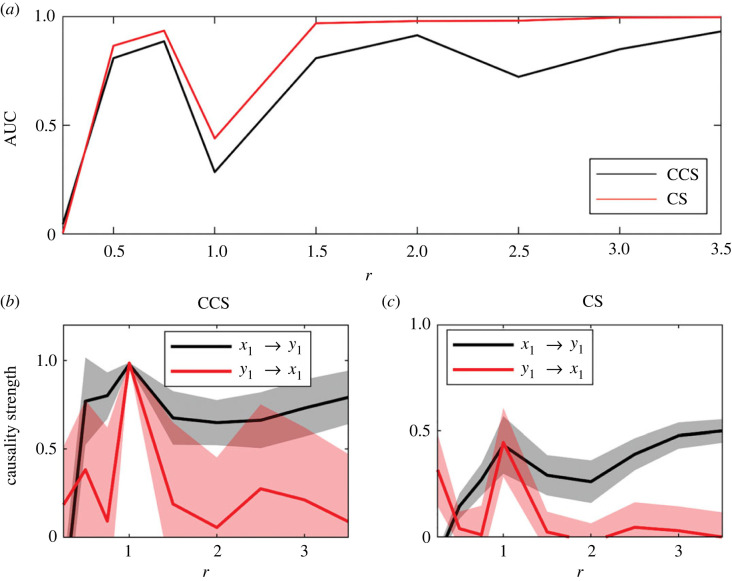
Causality strength detected using CCS and continuity scaling methods for increasing time series length (*L*). Results were averaged over 1000 simulations with random initial conditions for each value of *r*. (*a*) Variation AUC as *r* increases. Causality strength detected by the (*b*) CCS and (*c*) CS methods.

The convergence of both causality detection methods as a function of time-series length is shown in figure 17.

Both methods detect bidirectional relationships in the Angat dam system (table 3), which is incorrect. However, in both cases, the main direction of causality was detected correctly (figure 17*a*). For the case of the Ipo dam, both methods detect a correct causal relationship. For the chemostat data, only the CS method correctly captures the bidirectional causality, and the detected causality strength is of the same order in both directions, C(*Brach*. → *Chlor.*) ≈ C(*Chlor*. → *Brach.*) (figure 17*c*). Using the CS method, causality strength converged to a relatively uniform value of 0.4 at *L* = 350 (figure 17*c*), which is a moderated time-series length considering actual real-world experimental and observational databases.

## 5. Discussion

The CS method exhibited the best performance for all systems under study. The CS method reaches its maximum efficiency value at *L* ≈ 150 when results from the LM were analysed. However, its efficiency was lower than the CCS method when at coupling strengths of *C* ≈ 3 (figure 7*a*). Almost complete synchronization was observed for this value of *C*, and the time series were almost identical. This is reflected in the correlation value being close to 1 and a marked increase in MI (figure 7*b*,*c*).

An important question is how we can distinguish between unidirectional, bidirectional, or asymmetric bidirectional causality. This is studied in figure 9. From these results, it is evident that the CS method is superior to the CCS method in the general bidirectional case, which can be observed directly in figure 9. This superiority is also evident in the smaller RMSE values in table 1 and the larger AUC in the same table. The RMSE values measure the ability of the methods to recreate the real causal relationship, while the AUC measures the ability to detect the correct causal links (independently of the predominant direction).

For the RL system (figure 12), CS method exhibits a considerably higher efficiency, particularly for short time series, where the method reached its maximum AUC at *L* ≈ 200.

In practical applications, an important question is: how do we know if the time-series length is long enough to apply some specific methodology? The answer depends on the variability that time series exhibits at different time scales which is particular to each system, and also on the performance that each method has in these specific cases. Before applying any causality detection methodology, a previous sensitivity analysis should be conducted and its convergence as a function of time-series length (*L*) should be checked. This is observed for the simulated systems in figures 6*b*,*c* and 12*b*,*c* and also in the real-world systems under consideration figure 17). In the latter results, we observed that for short time-series lengths, there are significant variations in the causality values (figure 17*a*,*c*), reaching relatively stable causality strength values as the length increases, specially for the CS method. For this latter method, we observe a level of convergence for the Ipo dam system starting at *L* ≈ 2000, which was not observed in the results of the CCS method.

Synchronization has been identified as a problem in causality detection [26,27]. It has been suggested [29] that the CCS method has a higher efficiency than the CCM in systems susceptible to synchronization. In figure 13*a*, we observe a low efficiency of the CCS method (AUC ≤ 0.7 for all values of the coupling strength *C*) in the RL system (*a* = 6), where generalized synchronization starts at *C* ≈ 2. Even before synchronization, the efficiency of CCS was less than the efficiency estimated when the CS method was applied. When these results are compared with the Lyapunov exponents (LE) reported in [42], a similar behaviour of the AUC and the distribution of exponents is observed. When LE are positive, greater efficiency is achieved for both methods. The same was observed in simulations with *a* = 10. MI can be interpreted as an indicator of synchronization [52]. We observed that under conditions of ‘high’ MI, a lower efficiency in causality detection can be observed (figures 7 and 13). However, given that the values estimated for mutual information are relative to each particular system, the designation of ‘high value’ given a single realization of the system requires the introduction of some kind of suitable normalization.

Regarding the causality strength, it is clear that the CS method performs better than the CCS method in the following aspects that emerged after the comparison of figure 8 and figure 14. First, it detects values close to 0 in the absence of causality (not so the CCS method). Second, from figure 8, the causality strength detected by the CS method increases progressively with the actual coupling parameter (*C*) up to *C* ≈ 2, which is not the case for the CCS method that quickly saturates at 1. The same can be observed in the results of figure 9, where it is clearly observed that the CS method recreates the gradient in the causality strength difference, while the CCS method does not. Accordingly, our recommendation is to analyse both the statistical significance of the causal link and the causality strength ratio *C*(1 → 2)/*C*(2 → 1) instead of directly studying the value detected by each method.

When the effect of the SNR on the methods' abilities to detect causality was analysed, a superior performance for the CS method was observed in all scenarios (figures 10 and 15). Results obtained for the LM showed low efficiencies for both methods when the noise level was high (SNR < 10, figure 10). The results were different for the RL system: the CS method showed a similar efficiency for all noise levels (AUC ≈ 0.9) and convergence of causality strength at SNR ≈ 20. For the same system, the efficiency of the CCS method decreases as noise decreases. This means that the CS method was more robust in the presence of noise than the CCS method.

Biological and ecological systems can develop feedback loops or linear causal networks [33,34]. The simplest causal network topologies are shown in figure 1. Results observed in figure 11 indicate that both methods can accurately distinguish between direct and indirect causality.

Time series in natural sciences and geophysics are characterized by multiple time scales of variability, which means that its variance is concentrated on different frequency bands. In these research areas, the use of statistical methods (e.g. wavelet coherence [53]) to detect and quantify synchronous oscillations, and accordingly, they infer causality based on the degree of coupling between both time series. This statistically based approach can detect artificial couplings and, therefore, spurious causality. By contrast, for CCS and CS methods, synchrony makes the detection of causality more difficult, and both methods fail in the presence of generalized synchrony. In brief, synchrony should be considered as a factor that enhances the possibility to detect causal links that do not exist. Our results show that for RR systems with a large difference between their characteristic frequencies, causality tends to be detected from the low-frequency to the high-frequency system in circumstances where the simulated experiment is designed with a causal link in the opposite direction (as we can observe at *r* = 0.25 in figure 16). This bias, favouring causality from slow to fast variables, has previously been observed in [54]. Overall our results show a superior performance of the CS method, with an AUC ≈ 1 starting at *r* = 1.5. Our results suggest that in experimental time-series exhibiting significant seasonality, causality analysis should be complemented by other statistical analysis. One possible option is to analyse the statistical significance of the causality by studying surrogates with the same main frequency but applying the stationary bootstrap to the fluctuations (considering that experimental time series with marked seasonality are usually composed of slow, or seasonal and fast, time scales); this should test whether the fast variable could be caused by another variable with the same main frequency [55]. The other option is to extract the main frequency content from the slow variable and apply the causality detection methods and surrogate analysis to the fluctuations.

## 6. Summary and conclusion

Our results show that, when detection of causality in simulated and real-world time series under different scenarios was conducted, the CS method appears to be more robust and efficient than the CCS method. A better performance of the CS method in comparison with the CCS method was observed for all analysed time series, including series as short as *L* = 150 for which an AUC ≈ 0.95 was obtained. In general terms, the minimum time-series length necessary to detect causality depends on the system intrinsic dynamics and on the selected method. An appropriate use of causality detection methods should include a test to check convergence of causality strength as a function of time series length *L*. Standardization of both time series before applying the CS method is also recommended, to make results independent of geometric transformations and from the units under which time series were measured. The CS method clearly outperforms the CCS method when the strength and direction of causality in LMs with bidirectional coupling was analysed (RMSE = 0.297 and 0.450, respectively). The ability of both methods to detect the right direction of causal links can be evaluated checking the statistical significance with their *p*-values. It was observed that the CS method was more efficient in detecting causality than the CCS method, achieving the former higher AUC values for equal time-series length and parameter configuration than the latter method (AUC = 0.962 and 0.553, respectively).

Neither method was able to detect causality when generalized synchronization dominates the dynamics of the system under study and using the mutual information as an indicator of synchrony. In addition, both methods did not perform well in determining the level of causality strength. Therefore, causality ratios *r*_*c*_(1 → 2) = *C*(1 → 2)/*C*(2 → 1) should be calculated, as they provide an indication of the relative causality strength. The addition of increasing noise levels to simulated series did not change previous conclusions, because the CS method showed the highest efficiency in causality detection under all configurations in comparison with the CCS method. When a remarkable difference between main frequencies of interacting time series was detected, both methods failed in detecting causality. For example, when *r* = 0.25 both methods detected a causal link going from the time series dominated by a low-frequency component to the series dominated by a high-frequency component. This latter result is essentially wrong because simulated time series emerged from an experiment designed with a causal link in the opposite direction.

Using real-world time series from the chemostat experiment (*Brachionus*/*Chlorella*) only the CS method was capable to properly detect bidirectional causality, but fails as the CCS method, spuriously detecting causality from the dam water level to the amount of rainfall in Angat (table 3). Most probably, as the water dam level time series is characterized by a marked periodic component, not observed for the rainfall time series which is more intermittent (figure 4*a*), the difference between its main frequencies could be the cause of this failure as demonstrated above using simulated series. Time series dominated by low-frequency components are ubiquitous in the real-world, and climatic/oceanographic time series and population abundance time series from diverse marine ecosystems are only a few examples of series for which low-frequency components tends to dominate. When causal links are detected using this latter type of series, our results and warnings should be considered, otherwise the rate of spurious detection of causal links can becomes higher than it should be if detection methods were not biased.

In conclusion, the process of development and application of causality detection methods is still far from achieving a mature state, which makes it, nowadays, not possible the use of a unique and easy recipe of widespread acceptance within the scientific community that allows a reliable detection of causal networks. Moreover, both methods can fail even when recommendations given above were considered. General guidelines given here must be complemented with a deeper knowledge of underlying mechanisms involved in the generation of causal links.

## Data Availability

Code for the continuity scaling method is available at https://github.com/bianzhiyu/ContinuityScaling. Code for convergent cross sorting method is available at https://github.com/lbreston/CCS. Simulation data are available upon request to pabcornejo@udec.cl.
